# An Unusual Case of Fatal Thoracoabdominal Gunshot Wound without Diaphragm Injury

**DOI:** 10.3390/diagnostics12040899

**Published:** 2022-04-04

**Authors:** Sara Sablone, Valeria Lagona, Francesco Introna

**Affiliations:** Section of Legal Medicine, Department of Interdisciplinary Medicine, University of Bari, Piazza Giulio Cesare 11, 70124 Bari, Italy; v.lagona@studenti.uniba.it (V.L.); francesco.introna@uniba.it (F.I.)

**Keywords:** thoracoabdominal gunshot wound, diaphragm injury, inferior cava vein lesion, terminal ballistic, forensic pathology, autopsy

## Abstract

In case of thoracoabdominal gunshot wounds (GSW), diaphragmatic lesions are common autopsy findings. In these cases, the bullet’s path involves both the thorax and the abdomen, so the diaphragm (the muscle that separates the two cavities) is frequently damaged. In the present report we illustrate a very unusual autopsy finding, came up after a man was shot twice and affected by a lethal thoracoabdominal gunshot wound. In particular, as expected based on CT scans, the corpse exhibited a thoracic-abdominal path and a retained bullet in the abdomen, but no diaphragmatic lesions or hemorrhagic infiltrations of this muscle have been detected during the autopsy. After a scrupulous examination and the section of all the organs, the intracorporeal projectile’s path was reconstructed, inferring that the thoracoabdominal transit of the bullet extraordinarily had occurred in correspondence of the diaphragmatic inferior vena cava’s ostium, thus exploiting a natural passage without damaging the diaphragmatic muscle.

## 1. Introduction

The effects of a projectile in the body may depend on various factors: velocity, entrance profile, bullet diameter, bullet path inside the body, and biological characteristics of the affected organ [1]. Gunshot wounds (GSW) both affecting the thorax and the abdomen are strongly suggestive of diaphragm damage, until proved otherwise [2]. Moreover, when the bullet causes great vessels’ injury, it usually leads to instantaneous death due to profuse hemorrhage [3]. In particular, inferior cava vein (ICV) injuries are very rare, but mortality is high, especially if supra or retro-hepatic lacerations are associated [4].

The purpose of this report is to illustrate the case of a man with a penetrating thoracoabdominal GSW and a perforating one at the level of the lower abdomen area. The retained bullet causing the thoracoabdominal GSW had a very unusual path: the bullet extraordinarily has gone through the diaphragmatic ICV’s ostium, exploiting a natural path without damaging the diaphragmatic muscle.

To date, this finding has never been reported in the relevant scientific literature. This event is peculiar if we consider that diaphragmatic injuries appear in 1% to 7% of patients with significant blunt trauma and in 10% to 15% of those with penetrating wounds, such as stab wounds and GSWs [5]. In particular, a gunshot wound anywhere within the chest or abdomen puts the patient at very high risk for penetrating diaphragmatic injury because of potential prolongation of the projectile’s travel within the body [5]. Moreover, the scientific literature encounters more diaphragmatic injuries by left-sided shooted bullets (such as in the case presented) since there are more right-handed assailants [5,6].

## 2. Case Report

A 27-year-old man presented at the Emergency Department with a penetrating GSW on the left subclavian region, and two perforating GSWs of the abdomen lower quadrants, linked by a hypodermis path. The patient died from hemorrhagic shock just after he was rushed to the hospital.

A complete autopsy was ordered by the judicial authority to verify the cause of death and to detect terminal ballistic characteristics.

### 2.1. The Corpse External Examination

In the left subclavicular region, there was a skin break in continuity, with irregular circular shape and frayed, introflexed edges of 0.4 cm in diameter. This injury was also surrounded by a brownish, annular and bruised-excoriated border, with dimensions of 1.6 × 1.3 cm with major transverse axis.

**Figure 1:**
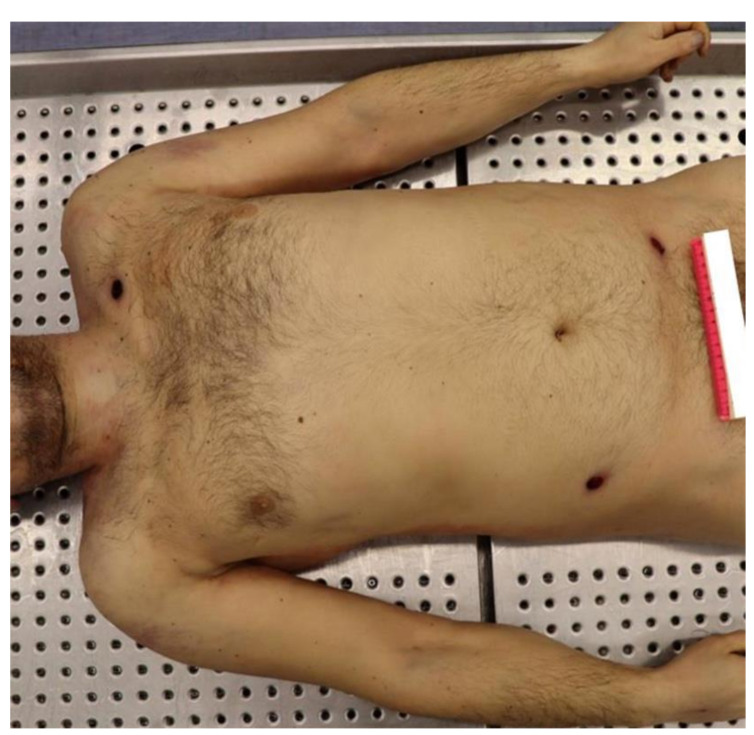
The corpse on the autoptic table at the moment of the external examination. The inlet and outlet ports of the perforating GSW are clearly visible, and so does the inlet port of the retained bullet.

In the right hemi-abdomen, between the flank and the ipsilateral iliac fossa, there was another skin break in continuity of 0.7 cm in diameter, with an irregularly circular shape, frayed edges and an ecchymotic-excoriated border with brownish-red colour. At last, in the left iliac fossa, there was a break in continuity of the skin, consisting of an irregular oval-shaped injury of 1 × 0.3 cm with a transverse major axis, with an epidermal flap on the medial extremity and a lateral ecchymotic-excoriated border of a reddish color (Figure 1).

### 2.2. Post-Mortem Instrumental Investigations

**Figure 2:**
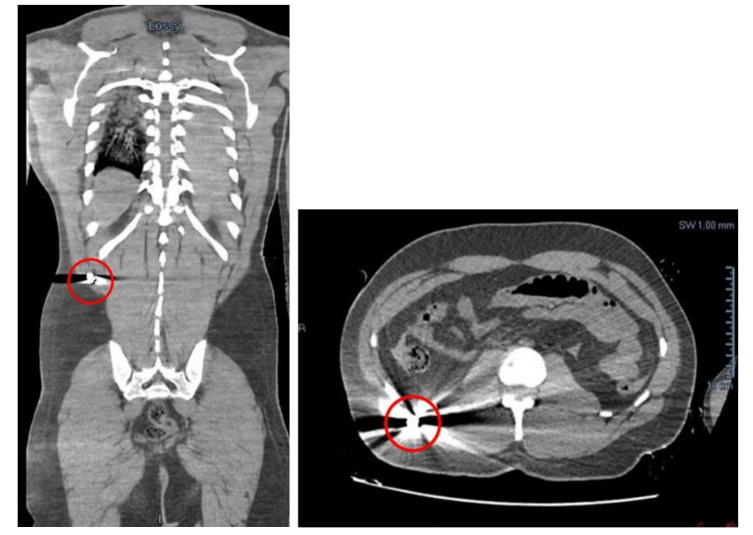
The retained bullet in the thickness of the posterior abdominal wall.

Before the autopsy, a full-body CT was performed. It was found a massive left hemothorax and free air at the level of the left hemithorax itself and of the homolateral subclavear and pectoral regions. More free air was found in the mediastinal area and small bubbles in the ventricles were detected. There were also an haemopericardium (with a thickness of 2 cm) and an hemomediastinum. Moreover, little bubbles were found in the hepatic parenchyma and in the left anterior abdomen. A liquid effusion was detected in the subcapsular area of the liver, in the Morrison’s pouch, in the subcapsular region of the right kidney and in the pelvic area. In the right lateroposterior extra-abdomen area, in the homolateral external oblique muscle and on the subcutaneous side, there was a small and hyperdense image, identifiable in a projectile’s ogive of 9 mm caliber. The external oblique muscle has traumatic lesions (Figure 2). In the end, a projectile’s path has been appreciated in the subcutaneous region of the antero-inferior abdominal surface, with a cutaneous entrance GSW on the right anterolateral abdominal surface and an exit one on the left anterolateral side.

### 2.3. Autopsy Findings

**Figure 3:**
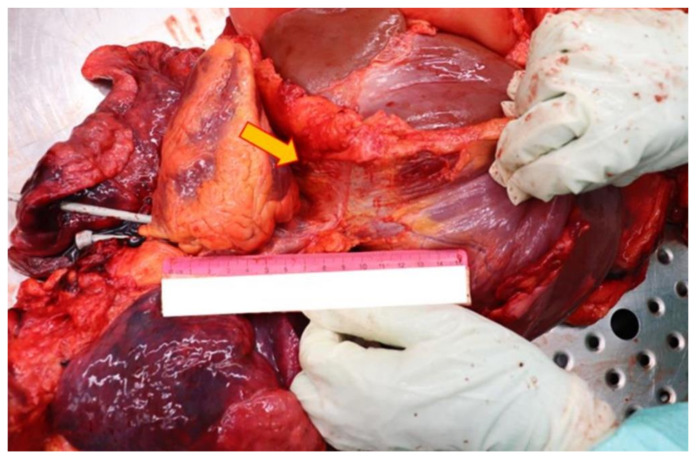
In this picture, it is plainly visible the diaphragm, with its subcardiac part (yellow arrow), without any lesion.

**Figure 4:**
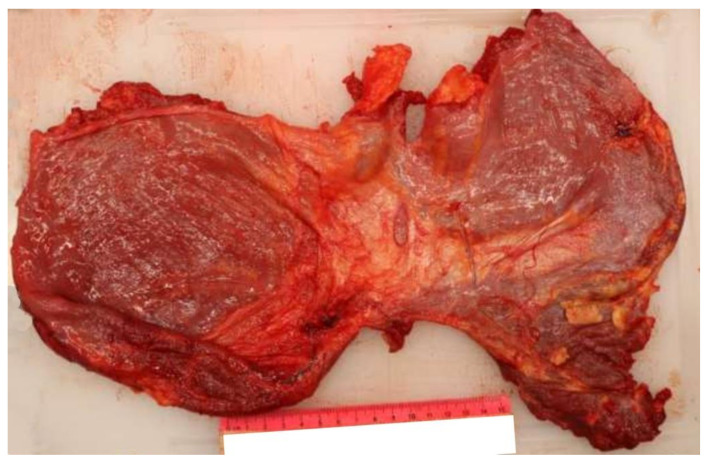
A completely intact diaphragm: a very rare necropsy finding in a thoracoabdominal GSW.

Thanks to the further complete autopsy examination, it has been possible to better assess the lesions provoked by the retained projectile. Also, it turned out that the penetrating bullet’s channel happens to be unique: the entry hole was located at the level of the left subclavear region and the projectile was found in the abdominal area, but the diaphragm wasn’t damaged at all. To better comprehend the reason of this unusual finding, the complete path was reconstructed: after having transfixed the subclavear region’s cutis and subcutis, the projectile first pierced the muscles of the second intercostal space and after the anterior surface of the left lung’ superior lobe. Then the bullet penetrated the superolateral surface of the left pericardium, the left auricle, the anterolateral surface of the pulmonary trunk and the right atrium. Oddly the projectile perforated the ICVand then passed through its diaphragm ostium, leaving the muscle completely intact (Figure 3 and Figure 4). 

**Figure 5:**
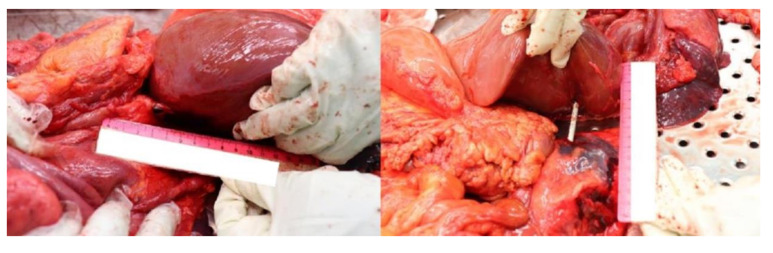
Thanks to the use of a probe, it has been possible to point out the projectile’s path through the ICV’s diaphragmatic ostium (left picture). The bullet also transfixed the infero-posterior surface of the liver, the right kidney’s capsule and its anterosuperior surface (right picture).

After that, the bullet transfixed the inferoposterior surface of the liver, the right renal’s capsule and its anterosuperior surface (Figure 5). 

**Figure 6:**
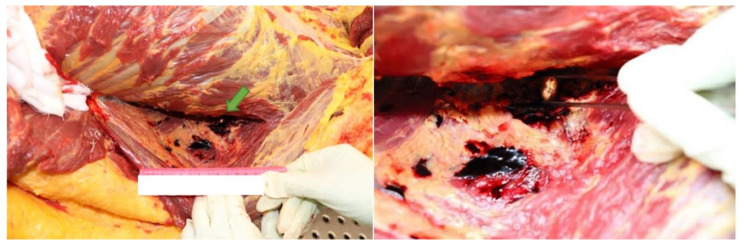
The retained 9 mm bullet (green arrow) found embedded between the internal oblique muscle and the transversus abdominis muscle.

At autopsy, the bullet was found embedded between the internal oblique muscle and the transversus abdominis muscle (Figure 6).

For what concerns the antero-inferior abdominal surface’s GSW, it showed a hypodermic path with no muscle or internal organs’ involvement and with very few signs of hemorrhagic infiltration.

Eventually, the cause of death was attributed to the hemorrhagic shock caused by the thoraco-abdominal GSW, since the other path did not produce any fatal injury.

### 2.4. Ballistic Findings

The retained bullet was a yellow, not deformed, metallic ogive, with a weight of 6030 g. At the epimicroscopic examination, there were no rifling marks. The projectile has a 9 mm diameter and was a.380 auto, fired at a distance of 3–5 m. Its morphological and dimensional characteristics allowed us to assert that the gun used did not have rifling grooves on the barrel inner surface. It is therefore hypothesized that the weapon was a worn-out gun (so with the leveling of the rifling marks), a prop gun, or a modified toy gun (with recanalization of the barrel).

## 3. Discussion and Conclusions

Generally, a bullet that penetrates the human body travels in a straight line and either goes through it leaving the body, or dissipates its kinetic energy and stops inside soft tissues [7]. In the latter case, the bullet can generally be found on radiological examination.

Of all GSWs, chest and abdominal ones have a higher mortality rate if compared to other ones. In particular, chest GSWs have a mortality rate which varies from 14.3% to 36.8% [8] because of the risk to produce heart damage (24.5%), hemothorax (59.9%), pneumothorax (37.9%), lungs’ lacerations (79%), bleeding of intercostal/internal mammary vessels (65%), and subclavian arteries’ lacerations (29%). Instead, the abdominal ones present a fatality rate of 10% and they are frequently characterized by peritoneal penetration, solid organs’ injuries (such as liver, kidney, or spleen’s injuries), and retroperitoneum structures’ damage [9].

As set out in this case report, the 9-mm projectile has penetrated both the thorax and the abdomen of the patient, and then it was embedded in the abdominal muscular layers. Along its path inside the body, the bullet has pierced the anterior surface of the left lung’s superior lobe, the superolateral surface of the the left pericardium, the left auricle of the heart, the anterolateral surface of the pulmonary trunk, and the right atrium. Subsequently, the projectile pierced the intrahepatic tract of the ICV. Eventually, before being embedded between the right internal oblique muscle and the transversus abdominis muscle, the bullet transfixed the inferoposterior surface of the liver, the right renal’s capsule and its anterosuperior surface.

Thanks to the ballistics analysis, it is possible to assert that the bullet caliber was compatible with the path’s size (the permanent cavity), thus confirming that the projectile did not fragment when it passed through tissues. The bullet caliber also appeared compatible with the ICV’s diaphragmatic ostium diameter. Moreover, it was possible to establish that the bullet had a subsonic speed.

During its intracorporeal travel, the bullet’s kinetic energy undoubtedly generated a temporary cavity, caused by the radial transfer of energy from the moving bullet to the surrounding tissues, piled in front of it. Furthermore, the temporary cavity extent estimation has been possible even through the tissues’ gross anatomical observation by evaluating the path’s collateral hyperemia areas.

To the best of our knowledge, there are no literature reports demonstrating the regular correlation between bullet physical characteristics (particularly the bullet velocity) and the bullet penetration’s effects on the GSW human victims’ bodies. Indeed, according to the energy conservation law, the temporary cavity formation depends on the bullet’s energy adoption by tissues. Thus, the bullet-tissue interaction (i.e., the forces operating at the energy exchange) appears to be particularly crucial and affected by many factors, such as the bullet design (shape, sectional density, velocity at the time of impact) and the type of tissue at the interface (solid, fluid, gaseous) with its specific elastic properties [10]. In this regard, a few regularities can be observed, and solely under laboratory conditions with extreme simplifications (e.g., shooting at homogeneous media or homogeneous tissues and organs) [11,12,13,14]. The obtained results are not always reproducible and cannot form the basis for conclusions about the effects of GSWin real conditions (crimes, hunting) [10,15].

Generally, the described energy transfer causes a sudden increase of pressure in tissues that may lead to their damage. It seems to be particularly frequent when the GSW involves organs devoid of elastic elements, such as parenchymal organs (e.g., liver, kidneys). On the contrary, elastic tissues (e.g., muscles, lungs) are largely resistant to this type of damage thanks to their high flexibility and high rupture strength [16,17,18].

In conclusion, the wound ballistics literature seems to contain several misconceptions about the physical effects of penetrating projectiles in tissue and tissue simulants. Sometimes, these can adversely affect the proper management and the forensic interpretation of gunshot injuries.

The dynamic phenomenon of temporary cavitation is, and should continue to be, extensively reviewed for what concerns its impact on wound production and the associated controversy surrounding its consequences in soft tissue wounds [15]. Part of this controversy emanates from the misinterpretation of experimental data regarding the magnitude of the temporary cavity induced by high-velocity projectiles and the different conceptions of the tissue response to cavitation [15]. However, as stated above, many factors may strongly influence the energy transfer characteristics affecting both the temporary cavitation and the size of the permanent wound channel.

Thus, the case presented demonstrates how, despite the formation of a temporary cavity, the projectile’s harmful effects on deep human tissues are not always precisely predictable.

In the presence of a fatal thoracoabdominal GSW with signs of tissue damage around the permanent cavity, regardless of the bullet velocity, we would have expected to find diaphragm devastation or, at least, discontinuation. Instead, having the bullet exploited a natural path (the diaphragmatic ICV’s ostium), the complex phenomena underlying terminal and wound ballistics have allowed encountering an extremely rare autopsy finding in a fatal thoracoabdominal GSW, i.e., a perfectly intact diaphragm.
